# Molecular Detection and Genetic Variability of *Hepatozoon canis* in Golden Jackals (*Canis aureus* L. 1758) in Serbia

**DOI:** 10.3390/biology13060411

**Published:** 2024-06-04

**Authors:** Milica Kuručki, Ratko Sukara, Valentina Ćirković, Duško Ćirović, Snežana Tomanović

**Affiliations:** 1 Faculty of Biology, University of Belgrade, 11000 Belgrade, Serbia; dcirovic@bio.bg.ac.rs; 2 Group for Medical Entomology, Centre of Excellence for Food- and Vector-Borne Zoonoses, Institute for Medical Research, National Institute of Republic of Serbia, University of Belgrade, 11129 Belgrade, Serbia; ratko.sukara@imi.bg.ac.rs (R.S.); valentina.cirkovic@imi.bg.ac.rs (V.Ć.); snezanat@imi.bg.ac.rs (S.T.)

**Keywords:** golden jackal, *Hepatozoon*, Serbia, *Canis aureus*

## Abstract

**Simple Summary:**

In addition to infecting domestic dogs, *Hepatozoon canis* affects a large number of wild canids, such as foxes, jackals, and wolves, that can serve as reservoirs for this pathogen. In this study, we focused on the prevalence of *H. canis*, its genetic diversity, and its distribution in golden jackals on the territory of Serbia. At the same time, this is the first study in which a high genetic diversity of four sequence types (S4–S7) of *H. canis* has been found in golden jackals. In addition, a high prevalence rate was found, with 78.95% of animals testing positive for *H. canis*. Genetic analysis revealed variability at four positions, resulting in four different sequence types in golden jackals. Animals can become infected through the ingestion of infected ticks or by consuming infected prey, as well as transplacentally, from mother to offspring; what it could testify to 6/15 positive juvenile jackals. Additional research is crucial for elucidating transmission mechanisms, pinpointing potential sources of infection, and comprehending the ramifications of this pathogen for wild carnivores.

**Abstract:**

*Hepatozoon canis* is a protozoan tick-borne parasite infecting domestic and wild canids, including foxes, wolves, and jackals. It is mainly found in dogs but has also been detected in several wild carnivores, including foxes, wolves, and jackals. Host transmission primarily occurs through the ingestion of infected ticks, typically *Rhipicephalus sanguineus*, with documented instances of transplacental transmission from infected females to cubs. In Serbia, the golden jackal is common throughout the country, and its population has increased in recent years. Previous research has documented the presence of several vector-borne pathogens in the jackal population in Serbia, so we conducted this study to determine the presence, prevalence, and genetic variability of *H. canis*. Over eleven years (2010–2020), 114 animal samples were collected from 23 localities in Serbia. A total of 90/114 (78.95%) jackals were positive for *H. canis*, and they came from 22 localities. Among 15 juveniles, almost half (6/15 (40%)) tested positive for *H. canis*. In addition to the high prevalence, high genetic variability of the pathogen was also found. According to the mutated positions, four sequence types (S4–S7) of *H. canis* were determined. Based on our earlier research on the grey wolf and on this study, it can be observed that various sequence types of *H. canis* circulate within wild canid populations in Serbia. The prevalence of *H. canis* infection in wild carnivores raises significant concerns for wildlife conservation and animal health. Infected animals may act as reservoirs for the disease, posing a potential risk to domestic animals by acting as a source of infection.

## 1. Introduction

The golden jackal (*Canis aureus* Linnaeus, 1758) is currently considered one of the most thriving mesocarnivore species, and its population has undergone significant range expansion in the last several decades. Although its native distribution was originally restricted to southern Asia, as well as southeastern Europe, today, golden jackals can be found throughout the entirety of eastern and central Europe and their territory is rapidly expanding towards western and northern Europe [1,2,3]. However, the Balkan Peninsula is often considered a center of species distribution, where the golden jackal represents the second most common canid species [1,4]. Previous studies have suggested a wide range of factors influencing jackal territory expansion, among which the increased availability of food waste from humans, significant land-use changes, climate change, and the absence of wolves are often considered the most important [5]. Therefore, due to their high adaptability and opportunistic diet, jackals are capable of successfully exploiting a wide variety of habitats, including human-modified ecosystems [6]. Moreover, according to population densities, it has been shown, especially in the Balkans, that agricultural land is often the jackal’s preferred habitats [7]. In Serbia, jackals, as opportunists, have increased in number and are present on almost the entire territory. The population density is extremely high in the lowland areas, while it is much lower in the mountainous areas.

Although several studies have shown the jackal’s importance as a vector-borne pathogen reservoir [8,9], its epidemiological role remains poorly understood. Considering their significant territory expansion and ability to move over long distances within highly human-modified landscapes [5], jackals may represent potential reservoir hosts for several vector-borne pathogens.

*Hepatozoon* species, belonging to the apicomplexan parasite group, have the ability to infect a diverse range of animal species, including canids [10,11,12,13]. The species primarily found in the family Canidae is *Hepatozoon canis*. Its life cycle includes an invertebrate hematophagous vector as the final host and vertebrate hosts, including domestic dogs and wild canids, as intermediate hosts. The *Rhipicephalus sanguineus* tick is considered the primary vector of *H. canis* [14]. The pathogen has also been detected in other tick species, such as *Ixodes ricinus*, *Ixodes canisuga*, *Ixodes hexagonus*, *Ixodes ventalloi*, and *Dermacentor reticulatus* [15]. However, the role of these tick species in *H. canis* transmission is not clearly understood. A tick becomes infected during a blood meal when it ingests the gamont parasite. After ingestion, the gamonts develop into oocysts in the tick’s intestine. The oocysts mature and release sporozoites that migrate into the tick’s salivary glands. This infection survives throughout the developmental stages of the tick (larva, nymph, and adult) [11]. Vertebrates are infected by ingesting ticks containing mature oocysts of *Hepatozoon* species. Transplacental transmission of *H. canis* is also documented [16,17,18]. It is assumed that *H. canis* might be transmitted to domestic dogs from its wild canid relatives [11]. Considering that, in human-modified environments, jackals often approach human settlements, the interaction between domestic and wild canids may represent a particular level of risk concerning the emergence, spread, and maintenance of pathogens [9]. Therefore, golden jackals may play an important role in the epidemiology of diseases such as hapatozoonosis among European canids. However, the likelihood of *H. canis* infection in domestic dogs and wild canid populations can vary considerably, as they are found in different habitats where they may be exposed to a different spectrum of hematophagous vectors but also have different levels of susceptibility to the pathogen [19].

The importance of this study lies in the fact that it shows the golden jackal as a potential reservoir for *H. canis* and the possibility of the pathogen spreading to domestic and wild canids. The jackal population in Serbia is expanding both in size and range, and it is expected that this trend will continue in the coming years. Therefore, our main objective was to investigate the prevalence and genetic variability of *H. canis* infection in golden jackals in Serbia. Furthermore, we have analyzed the evolutionary relatedness of *H. canis* isolated from different host species in Serbia.

## 2. Material and Methods

### 2.1. Study Area and Sampling

During a period of eleven years (2010–2020), spleen samples from golden jackals (*Canis aureus*) were collected in cooperation with hunting associations in Serbia. Legally killed animals were dissected in the field, and, for the purpose of this study, the spleens of the golden jackals were collected. For each specific animal, information on the date of shooting, sex, age, and collection location was noted. After dissection, the samples were transported in portable freezers to the Faculty of Biology, University of Belgrade, where they were stored appropriately at a temperature of −20 °C until further analysis.

### 2.2. Molecular Detection and Characterization of H. canis

A small amount (up to 10 mg) of the golden jackal spleen samples was subjected to further processing. Samples were individually homogenized and underwent DNA extraction using the Gene Jet Genomic DNA Purification Kit (Fermentas, Thermo Fisher Scientific, Waltham, MA, USA). For *H. canis* detection, we amplified a 666-bp fragment of the 18S rRNA gene, using the set of primers HepF_for (5′-ATACATGAGCAAAATCTCAAC-3′) and HepR_rev (5′-CTTATTATTCCATGCTGCTGCAG-3′) [20], via conventional PCR. The reaction mix contained 24.75 µL nuclease-free water, 10 µL 5 X Green Reaction Buffer (7.5 mM MgCl_2_; pH 8.5), 1 µL dNTPs (10 mM), 0.250 µL Taq polymerase (5 u/µL, GoTaq G2 DNA Polymerase, Promega Corporation, Madison, WI, USA), 4 µL HepF_for primer (10 pmol/µL), 4 µL HepR_rev primer (10 pmol/µL), and 6 µL template DNA. Amplification conditions included initial denaturation at 95 °C for 2 min, then 40 cycles of denaturation at 95 °C for 1 min, annealing at 56 °C for 1 min, elongation at 72 °C for 1 min, and final elongation at 72 °C for 5 min in Eppendorf 5333 MasterCycler Thermal Cycler (Eppendorf, Hamburg, Germany) [21]. After electrophoresis, samples were visualized on a 2% agarose gel. Representative samples positive for *H. canis* were selected for further sequencing. The preparation of samples was performed using the above mentioned protocol for amplification of 666-bp fragment of the 18S rRNA gene, using a set of primers HepF_for and HepR_rev in 50 µL amplification volume. Electrophoresis was performed on a 2% agarose gel for all samples to evaluate success of amplification. Samples were sent for Sanger sequencing by a commercial service, Macrogen Commercial Laboratory in Amsterdam (The Netherlands). Initial sequence analysis was performed using the FinchTV (version 1.5.0) program.

### 2.3. Phylogenetic Analysis

The present study included all obtained 18S rRNA gene sequences, together with all sequences from Serbia, available in the GenBank (National Center for Biotechnology Information, http://www.ncbi.nlm.nih.gov/BLAST, accessed on 23 April 2024). One dataset of 77 *H. canis* 18S rRNA gene sequences, 35 obtained in this study and 42 previously published, was subjected to phylogenetic analysis. Sequence alignment was performed using the Clustal W algorithm implemented in MEGA X software [22] (Supplementary Table S1). Aligned sequences were manually inspected and edited to obtain optimal alignment with the maximum number of available sequences. The best-fit nucleotide substitution model for aligned sequences was determined by jModeltest 2.1.4 software, using all 88 proposed models [23]. The phylogeny was assessed using maximum likelihood inference implemented in MEGA X software [22]. The phylogeny was assessed using two different algorithms, including Bayesian and maximum likelihood inference. The Bayesian analysis was carried out by applying a Markov chain Monte Carlo (MCMC) run for 5 × 10^6^ generations with a sampling frequency of every 5000 generations and a burn-in of 25%, implemented in MrBayes 3.1.2 [24]. For maximum likelihood tree reconstruction, MEGA X software was used [22]. Sanger sequence editing, assembly, and generation of consensus sequences were conducted in FinchTV.

### 2.4. Statistics

**Figure 1:**
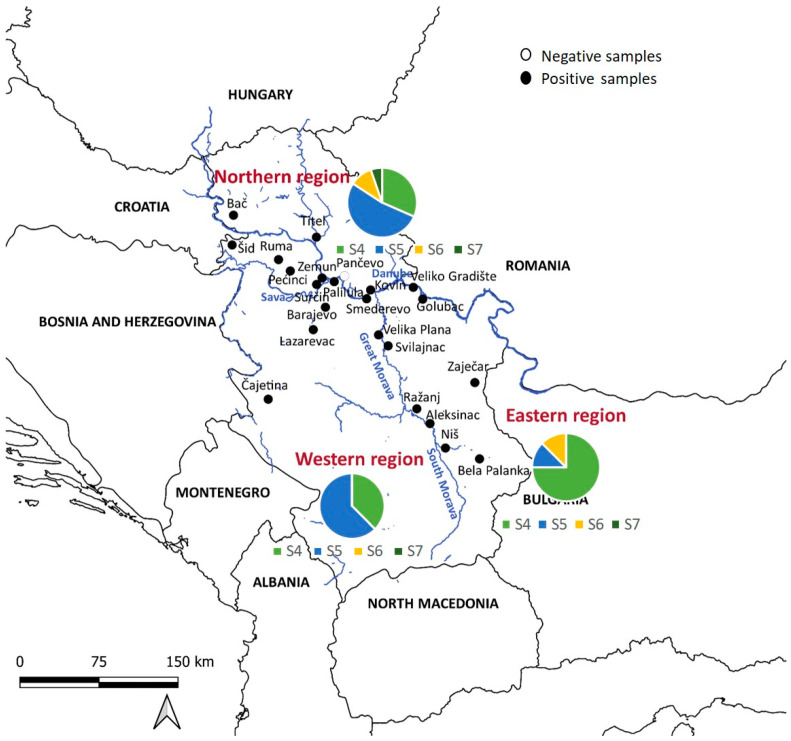
Distribution of golden jackal samples across Serbia’s regions. The country is delineated into three regions—northern, eastern, and western—by the Sava, Danube, Great Morava, and South Morava rivers. The pie charts show the distribution of sequence types S4–S7 in the northern (top), western (left), and eastern (right) parts of Serbia.

For statistical analysis, the golden jackal samples were divided into three subpopulations, the northern subpopulation, the western subpopulation, and the eastern subpopulation, depending on the location of the Sava and Danube rivers and the Great Morava and South Morava rivers, which form the border between these three regions (Figure 1). Briefly, ten localities were placed in the northern part, five localities in the western part, and eight localities in the eastern part of Serbia. Data were analyzed using R Software (version 4.2.2), with a significance level *p* < 0.05. The chi-squared test was employed to explain the differences in pathogen prevalence between sexes and ages (adults and juveniles). Jackals were classified according to their age into juveniles (<10 months) and adults (>11 months). We used the G-test to explain the difference in prevalence between the three defined regions.

## 3. Results

### 3.1. Demographic Characteristics and Regional Distribution of Sampled Golden Jackals

The golden jackals included in the study were sampled from 2010 to 2020 across twenty-three locations covering their distribution range in Serbia (Figure 1). A total of 114 spleen samples were collected, consisting of 50 females and 64 males. Among these, 15 (13.16%) were juveniles (3–7 months old) and 99 (86.84%) were adults (1+ year old). The sex ratio was 43.86% females and 56.14% males. The presence of *H. canis* was detected in 78.95% of the animals, with the prevalence higher in older animals. There was no significant difference in prevalence between sexes.

Infected animals were found in twenty-two out of twenty-three localities, with varying prevalence, ranging from 40% to 100% (Table 1). There was no statistical significance in prevalence between the northern (68.9%), western (95.6%), and eastern (84.8%) subpopulations.

### 3.2. Genetic Variability of H. canis

Sequencing analysis revealed variability in the *H. canis* sequences detected in jackals, with four distinct sequence types identified. The obtained PCR products were 666 bp in length on a 18S rRNA gene fragment (Supplementary File, Figure S1). A total of 35 representative PCR-positive samples (32 from adult animals and 3 from juveniles) were sequenced. All the obtained sequences were trimmed to remove primer sequences and low-quality regions prior to sequence analysis. Sequences that met quality standards (query coverage > 90% and identity > 99% with *H. canis*) were deposited in GenBank (PP711216–PP711250). 

**Figure 2:**
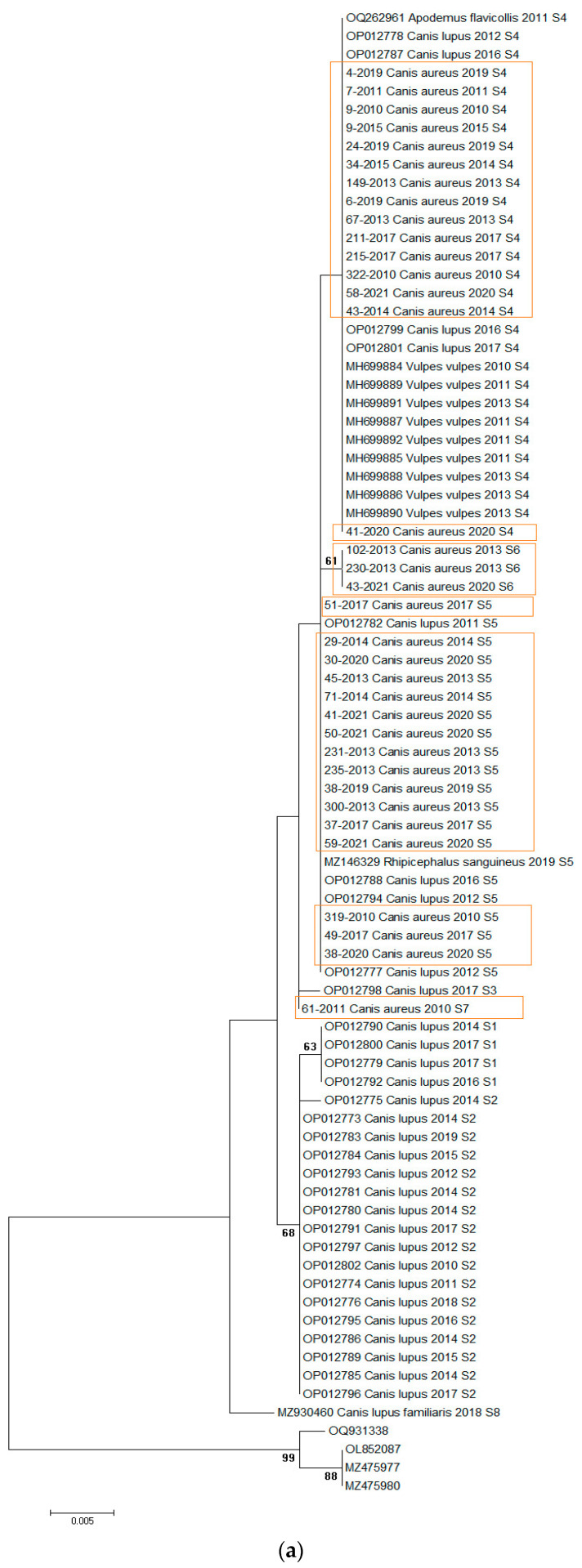

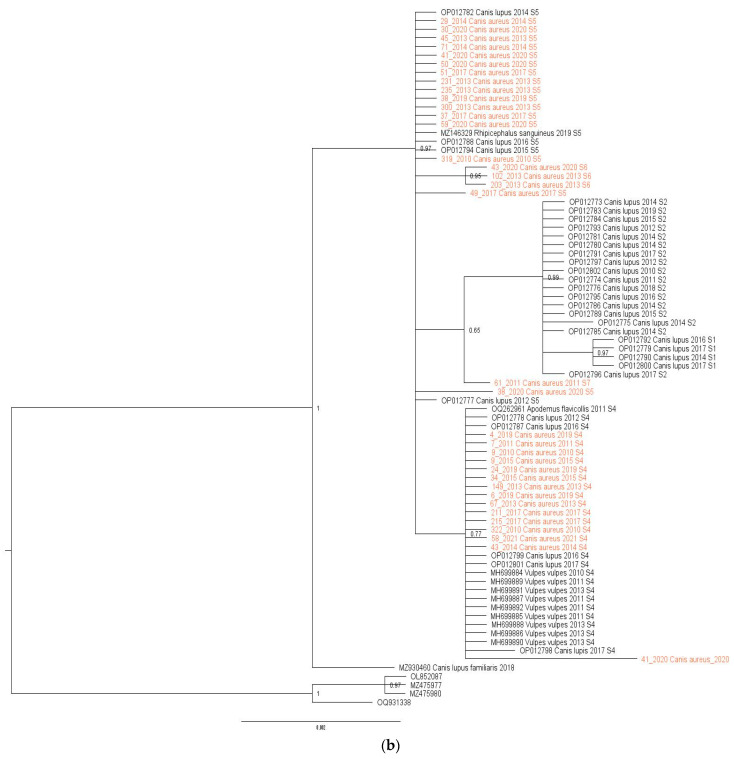
(**a**) Maximum-likelihood phylogenetic tree constructed using 77 18S rRNA fragment gene sequences of *H. canis*. The tree was rooted with corresponding sequences of *H. felis*. Bootstrap values ≥ 50 were shown. Numbers at the nodes indicate bootstrap support. Newly detected sequences were framed in orange. (**b**) Bayesian phylogenetic tree constructed using 77 18s rRNA fragment gene sequences of *H. canis*. The tree was rooted using four sequences of *Hepatozoon felis* (OQ931338, OL852087, MZ475977, and MZ475980). Numbers at the nodes indicate posterior probabilities. Newly detected sequences are labeled in orange.

Initial analysis of the dataset showed variability in *H. canis* sequences at three positions in jackals, allowing the identification of four sequence types (S4–S7). These sequences were analyzed alongside previously published sequences from Serbia, including those from wolves, foxes, dogs, *Apodemus flavicollis* mice, and *Rhipicephalus sanguineus* ticks. The combined dataset of 77 *H. canis* 18S rRNA gene sequences (35 from this study and 42 previously published) was subjected to phylogenetic analysis (Figure 2a). The alignment length was 567 bp, with all variable positions included (Table 2). All sequences retrieved from GenBank were from various animal species in Serbia (2010–2020, Supplementary Table S1).

The analysis revealed four sequence types (S4–S7) in jackals (Figure 2a, Table 2). We implemented sequence types derived from a prior investigation in wolves [21], wherein pathogen genetic diversity was notably elevated (S1–S5), subsequently identifying that S4 and S5 align with sequence types observed in jackals. Of the sequences identified, 14/35 (40%) were S4, 16/35 (45.7%) were S5, 3/35 (8.57%) were S6, and one was S7. Regionally, 19 sequences were from northern Serbia, 8 from western Serbia, and 8 from eastern Serbia. All sequence types were present in the north, three (S4, S5, S6) were present in the east, and two (S4, S5) were present in the west. S5 was predominant in the northern and western regions, while S4 dominated in the east (Figure 1).

The mean nucleotide distance of all sequences included in the study was 0.34% (range from 0 to 0.107%), while the nucleotide distance among *H. canis* sequences isolated from jackals was 0.12% (range from 0 to 0.34%) and from wolves was 0.32% (range from 0 to 0.86%). Maximum likelihood and Bayesian inference phylogenetic analyses of all sequences included in the study gave mostly congruent results (Figure 2a,b). Phylogenetic analysis revealed three large clusters (Figure 2a). The first clade (29/77, 37.66%) was heterogeneous, including sequences from grey wolves, red foxes, and jackals, and one from *A. flavicollis*, all S4. The second cluster (24/77, 31.17%) was diverse, containing sequences from grey wolves, jackals, and one *R. sanguineus* tick, predominantly S5, with three jackal sequences classified as S6. The third clade (21/77, 27.27%) was monophyletic, consisting of grey wolf sequences, mostly S2, with three S1. Three sequences did not fit into these clusters and were classified as S3, S7, and S8. Of note, the only exception between two phylogenetic trees was observed in the position of one sequence originating from a wolf (acc. no. OP012798). In a maximum-likelihood phylogenetic tree, this sequence was identified as the S3 sequence type, while in a Bayesian tree, it was classified into the S4 sequence type.

## 4. Discussion

In recent years, Hepatozoonosis, as a tick-borne disease, has shown increasing trends in canids all over the world. The tick *R. sanguineus* is recognized as the primary vector responsible for transmitting *H. canis* [14]. Additionally, this pathogen has been identified in other tick species, including *I. ricinus*, *I. canisuga*, *I. hexagonus*, *I. ventalloi*, and *D. reticulatus* [15]. It is noteworthy that all the mentioned tick species have been identified in Serbia, together with the different pathogens that they can transmit [25,26,27,28,29]. We hypothesized that jackals could play a significant role in the transmission of vector-borne diseases in different ecosystems. Previous studies have revealed a high prevalence and genetic variability of *H. canis* in red foxes and grey wolves in Serbia [21,30]. In the present study, we investigated the presence of *Hepatozoon* spp. in the golden jackal population in Serbia using molecular methods. Our results show a notably high prevalence of *H. canis* in golden jackals, with ~80% of animals being positive. This study also offers insights into the detailed genetic variability of this pathogen and its evolutionary relationships with other Serbian strains derived from the database.

The highest prevalence was seen in the western region (95.6%), followed by the eastern (84.8%), and the northern region (68.9%). The high prevalence in the western and eastern regions can be explained by the fact that the distribution areas of jackals and wolves in Serbia overlap in these regions. The former study, which investigated the rate of infection by *H. canis* in golden jackals in south-eastern and central Europe, showed a relatively high prevalence of this parasite (60.6%) [31]. Most of the samples (206/311) tested in this study originated from Serbia, with a prevalence of 67.5%, which could indicate that jackals could be an important potential reservoir host for *H. canis* in natural habitats.

In Serbia, a relatively high dispersion of *H. canis* was observed in different animal species, including the ticks *I. ricinus* [27] and *R. sanguineus* [32], dogs [33,34], red foxes [30], wolves [21], and *A. flavicollis* mice [35]. A high prevalence of *H. canis* was detected in golden jackals in Europe [33,36], as well as in wolves in Serbia (57.94%) [21], Germany (46%) [37], and Italy (75.8%) [38]. Compared to wolves and jackals, the prevalence found in red foxes varied significantly among European countries, being highest in Spain (100% and 91%) [39,40], Germany (77.6%) [41], Portugal (75.6%) [42], Serbia (61.2%) [30], and Austria (58.3%) [31]. Compared to jackals, previous studies registered a low prevalence of *H. canis* in domestic dogs (1.81%; 20.42%; 18.18%) [34,41,43] and cats (1.7%; 0.51%) [44,45]. 

Almost half of the juveniles (6 out of 15 individuals) tested in this study were positive for *H. canis*, suggesting that jackals can become infected at a young age, probably when they groom their fur and accidentally ingest an infected tick [46]. As already known, *H. canis* can be transmitted to dogs and other canids through the ingestion of an infected tick [11,14]. In addition, a high percentage of positive juveniles may also indicate a possible transplacental transmission from mother to offspring, which has already been described [16,17,18]. This vertical transmission route highlights the important role of the female that infects their offspring in an intrauterine manner.

Herein, phylogenetic analysis was performed to analyze the genetic variability of *H. canis* strains from jackals and to assess relatedness among *H. canis* 18sRNA sequences retrieved from Gen Bank and newly acquired sequences. The results of our study show a higher genetic diversity in the 18S rRNA gene sequences of *H. canis* originating from wolves compared to those from jackals. These differences may be attributed to factors such as host specificity and adaptation, host population dynamics, transmission dynamics, and the geographical specificities of the region. The higher diversity could point to wolves as primordial hosts sharing a longer evolutionary history of interaction with *H. canis*. The geographic areal and overlapping of wolf and jackal populations in Serbia differ between regions, leading to differences in exposure to different parasite strains or environmental conditions that favor the transmission of the parasite. The phylogenetic tree revealed the presence of three clusters. The vast majority of the sequences included in the study were from autochthonous canid hosts (*C. aureus*, *C. lupus*, *V. vulpes*), and individual sequences from *R. sanguineus* and *A. flavicollis* were also included. The first cluster contained 29 *H. canis* sequences detected in jackals, red foxes, wolves, and an *A. flavicollis* specimen, all belonging to the sequence type S4. The second cluster contained a total of 24 *H. canis* sequences from jackals and wolves and one sequence from an *R. sanguineus* tick, belonging to the sequence types S5 and S6. Only sequences isolated from wolves, assigned to the sequence types S1 and S2, formed the third monophyletic group. The sequence types S3, S7, and S8 were extracted as separate sequences on the cluster. Previous studies in Serbia have confirmed a high prevalence of *H. canis* in populations of red foxes (61.2%) [30] and grey wolves (57.94%) [21], while the results of the present study also show a high prevalence of *H. canis* in the population of golden jackals. These results could increase the risk of *H. canis* spreading from the sylvatic cycle to urban areas. Despite this assumption, the S8 sequence type (MZ930460), originating from a dog from Serbia [34], has not yet been detected in analyzed wild canids. Further large-scale studies are needed to prove whether wild canids can be the source of infection for domestic dogs, as very little data are available on the prevalence of *H. canis* in domestic dogs.

The phylogeny of 18S rRNA *H. canis* sequences detected in jackals showed the presence of four different sequence types (S4–S7) in conjunction with three variable sites, therefore indicating the high genetic diversity of *H. canis* within the population of jackals in Serbia. An interesting phenomenon is that sequence-type diversity was inversely proportional to prevalence in these regions. The greatest diversity was found in the northern region, where all four sequence types (S4–S7) occurred, then the middle level of diversity was found in the eastern region (S4–S6), and the least diversity was found in the western region (S4–S5). Similar findings were observed in the wolf population in our region [21]. Five different sequence types (S1–S5) were detected in the wolf population, of which S4 and S5 also occur in the jackal population in Serbia. The most frequent sequence type of *H. canis* detected in jackals was the S5 sequence type, followed by the S4 sequence type. According to the results of the phylogenetic analysis, the S4 sequence type was the most widespread and it was present in all three autochthonous canids (*C. lupus*, *C. aureus*, and *V. vulpes*) in Serbia. This sequence type was also found in *H. canis* isolated from yellow-necked mice (*A. flavicollis*). With all this in mind, we assumed that S4 was a basic sequence type, later diversified through a host change from the red fox to the jackal and wolf. The second most common sequence type was S5, found in *H. canis* isolated from jackals and wolves, as well as from the tick *R. sanguineus*. Based on these findings, it is reasonable to propose that *H. canis* S4 and S5 sequence types were the most represented in the studied population and the possible transmission route for these types could be explained through predation. The natural prey of wolves in Serbia usually comprises roe deer (*Capreolus capreolus*), wild boar (*Sus scrofa*), European hares (*Lepus europaeus*), and small rodents [47]. A recent study showed the presence of *H. canis* in the spleens of roe deer and chamois [15]. Compared to wolves, red foxes and jackals are omnivorous and highly adapted to coexist with humans in anthropogenic environments [48]. In such environments, the feeding habits of these species overlap to some extent, with small and medium-sized mammals (such as rodents and lagomorphs) representing very important food sources [6,49]. Accordingly, due to their diet, wild canids could become infected via prey, previously proven in some *Hepatozoon* species [50,51,52,53,54,55]. Sequence types S1 and S2 were represented in the third monophyletic cluster, which included only wolves. The finding of the highest genetic diversity of pathogens in wolves is because the population only declined due to organized poisoning after the Second World War, while the population of jackals on the territory of Serbia almost disappeared due to the planned poisoning of wolves [1]. After a period of stagnation, the jackal population began to increase in both number and range, and, since the beginning of the 21st century, its territory has expanded on a large scale [56,57]. It is estimated that the jackal population in Serbia today comprises more than 20,000 individuals, present on over 70% of the country’s territory and likely to expand further [58].

According to this study, the sequence types S1, S2, and S3 are characteristic only for wolves, and S6 and S7 only for jackals. The S4 sequence is characteristic for all three autochthonous canids in Serbia (*V. vulpes*, *C. aureus*, and *C. lupus*), and S5 is characteristic for the genus Canis (*C. aureus* and *C. lupus*). Further studies on the genetic diversity of *H. canis* in wild canid populations conducted in the larger geographic area could shed light on the phenomenon of the associations of pathogen strains to different vertebrate hosts. Previous studies have found a relatively low variability in *H. canis*, and it is still unclear whether there is a relationship between specific genotypes and host species or whether they circulate between different species [41]. The presence of identical pathogen genotypes in different animal species indicates possible direct transmission or horizontal transmission through these hosts [41,59].

## 5. Conclusions

Considering the limited data on *H. canis* in jackal populations, the results of this study represent a significant contribution to the overall knowledge about its prevalence and genetic diversity. The rapid territorial expansion of jackals, in combination with the registered high prevalence of this pathogen, raises concerns about possible transmission to both wild and domestic canids. Given the large number of infected puppies, the question arises as to the significance of the transplacental transmission of this pathogen or the accidental ingestion of ticks in young animals. Furthermore, the current study indicates a close association and the possible occurrence of a common transmission pattern between cohabiting populations of jackals and other wild canids: the red fox and the wolf. It is necessary to further investigate the role of autochthonous canids in Serbia due to the distribution of different sequence types of *H. canis*, to determine the pathways of pathogen transmission through nutrition and niche overlap.

## Figures and Tables

**Table 1:** Distribution of golden jackal (*Canis aureus*) samples based on sampling locality, gender, and prevalence of *Hepatozoon canis*.

Region	Locality (No. of Collected Samples)	*H. canis*-Positive	
		Male	Female
NORTHERN REGION	Kovin (3)	2/3 (66.6)	0/0 (0)
	Titel (1)	1/1 (100)	0/0 (0)
	Surčin (29)	10/13 (76.9)	11/16 (68.8)
	Bač (5)	1/3 (33.3)	1/2 (50)
	Palilula (2)	0/1 (0)	1/1 (100)
	Pećinci (13)	7/9 (77.7)	3/4 (75)
	Pančevo (1)	0/1 (0)	0/0 (0)
	Šid (2)	1/2 (50)	0/0 (0)
	Ruma (1)	1/1 (100)	0/0 (0)
	Zemun (1)	1/1 (100)	0/0 (0)
Total * (%)	**40/58 (68.9)**	**24/35 (68.57)**	**16/23 (69.57)**
WESTERN REGION	Barajevo (1)	0/0 (0)	1/1 (100)
	Lazarevac (2)	1/1 (100)	1/1 (100)
	Smederevo (10)	3/3 (100)	7/7 (100)
	Velika Plana (5)	2/2 (100)	3/3 (100)
	Čajetina (5)	3/4 (75)	1/1 (100)
Total * (%)	**22/23 (95.6)**	**9/10 (90)**	**12/13 (92.30)**
EASTERN REGION	Veliko Gradište (23)	11/14 (78.5)	8/9 (88.8)
	Golubac (1)	1/1 (100)	0/0 (0)
	Svilajnac (4)	2/2 (100)	1/2 (50)
	Ražanj (1)	0/0 (0)	1/1 (100)
	Zaječar (1)	1/1 (100)	0/0 (0)
	Aleksinac (1)	0/0 (0)	1/1 (100)
	Niš (1)	1/1 (100)	0/0 (0)
	Bela Palanka (1)	1/1 (100)	0/0 (0)
Total * (%)	**28/33 (84.8)**	**17/20 (85)**	**11/13 (84.62)**
**Ʃ**	**90/114 (78.95)**	**51/64 (79.69)**	**29/50 (58)**

*—x/n, where x refers to the number of positive samples and n to the total number of samples.

**Table 2:** Alignment of partial sequences of 18S rRNA gene (567 bp) and position of variable sites.

Sequence Type	Position of the Variable Site		
	85 bp	549 bp	550 bp
Consensus	A	T	A
S4			G
S5			A
S6		C	
S7	G		

Abbreviation. T—thymine, A—adenine, G—guanine, C—cytosine.

## Data Availability

The data presented in this study are available from the corresponding author upon request.

## Supplementary Materials

The following supporting information can be downloaded at: https://www.mdpi.com/article/10.3390/biology13060411/s1, Table S1: List of sequences used in the present study. Figure S1: *Hepatozoon canis* 18S rRNA partial gene amplification with the primers HepF_for and HepR_rev. Simbol: Ladder; 1, 2, 3, 4, 5 etc. samples from DNK; K- negative control; K+ positive control.

## References

[1] Arnold J., Humer A., Heltai M., Murariu D., Spassov N., Hacklander K. (2012). Current status and distribution of golden jackal *Canis aureus* in Europe. Mammal. Rev..

[2] Trouwborst A., Krofel M., Linnell J.D. (2015). Legal implications of range expansions in a terrestrial carnivore: The case of the golden jackal (*Canis aureus*) in Europe. Biodivers. Conserv..

[3] Hoffmann M., Arnold J., Duckworth J.W., Jhala Y.W., Kamler J.F., Krofel M. *Canis aureus*. The IUCN Red List of Threatened Species. 2018, E. T118264161A146194820.

[4] Kryštufek B., Murariu D., Kurtonour C. (1997). Present distribution of the Golden Jackal *Canis aureus* in Balkans and adjacent regions. Mammal. Rev..

[5] Lanszki J., Schally G., Heltai M., Ranc N. (2018). Golden jackal expansion in Europe: First telemetry evidence of a natal dispersal. Mamm. Biol..

[6] Penezić A., Ćirović D. (2015). Seasonal variation in diet of the golden jackal (*Canis aureus*) in Serbia. Mammal Res..

[7] Spassov N., Acosta-Pankov I. (2019). Dispersal history of the golden jackal (*Canis aureus* moreoticus Geoffroy, 1835) in Europe and possible causes of its recent population explosion. Biodivers. Data J..

[8] Ionică A.M., Matei I.A., D’Amico G., Daskalaki A.A., Juránková J., Ionescu D.T., Mihalca A.D., Modrý D., Gherman C.M. (2016). Role of golden jackals (*Canis aureus*) as natural reservoirs of *Dirofilaria* spp. in Romania. Parasites Vectors.

[9] Mitková B., Hrazdilová K., D’Amico G., Duscher G.G., Suchentrunk F., Forejtek P., Gherman C.M., Matei I.A., Ionică A.M., Daskalaki A.A. (2017). Eurasian golden jackal as host of canine vector-borne protists. Parasites Vectors.

[10] Smith T.G. (1996). The genus *Hepatozoon* (Apicomplexa: Adeleina). J. Parasitol..

[11] Baneth G. (2011). Perspectives on canine and feline hepatozoonosis. Vet. Parasitol..

[12] Solano-Gallego L., Baneth G. (2011). Babesiosis in dogs and cats—Expanding parasitological and clinical spectra. Vet. Parasitol..

[13] Alvarado-Rybak M., Solano-Gallego L., Millán J. (2016). A review of piroplasmid infections in wild carnivores worldwide: Importance for domestic animal health and wildlife conservation. Parasites Vectors.

[14] Baneth G., Samish M., Shkap V. (2007). Life cycle of *Hepatozoon canis* (Apicomplexa: Adeleorina: Hepatozoidae) in the tick *Rhipicephalus sanguineus* and domestic dog (*Canis familiaris*). J. Parasitol..

[15] Uiterwijk M., Vojta L., Šprem N., Beck A., Jurković D., Kik M., Duscher G.G., Hodžić A., Reljić S., Sprong H. (2023). Diversity of *Hepatozoon* species in wild mammals and ticks in Europe. Parasites Vectors.

[16] Murata T., Inoue M., Tateyama S., Taura Y., Nakama S. (1993). Vertical transmission of *Hepatozoon canis* in dogs. J. Vet. Med. Sci..

[17] Hodžić A., Mrowietz N., Cézanne R., Bruckschwaiger P., Punz S., Habler V.E., Tomsik V., Lazar J., Duscher D.D., Glawisching W. (2018). Occurrence and diversity of arthropod-transmitted pathogens in red foxes (*Vulpes vulpes*) in western Austria, and possible vertical (transplacental) transmission of *Hepatozoon canis*. Parasitology.

[18] Schäfer I., Müller E., Nijhof A.M., Aupperle-Lellbach H., Loesenbeck G., Cramer S., Naucke T.J. (2022). First evidence of vertical Hepatozoon canis transmission in dogs in Europe. Parasites Vectors.

[19] Margalit Levi M., Nachum-Biala Y., King R., Baneth G. (2018). A survey of *Babesia* spp. and *Hepatozoon* spp. in wild canids in Israel. Parasites Vectors.

[20] Inokuma H., Okuda M., Ohno K., Shimoda K., Onishi T. (2002). Analysis of the 18S rRNA gene sequence of a Hepatozoon detected in two Japanese dogs. Vet. Parasitol..

[21] Kuručki M., Tomanović S., Sukara R., Ćirović D. (2022). High Prevalence and Genetic Variability of *Hepatozoon canis* in Grey Wolf (*Canis lupus* L. 1758) Population in Serbia. Animals.

[22] Kumar S., Stecher G., Li M., Knyaz C., Tamura K., Mega X. (2018). Molecular Evolutionary Genetics Analysis across computing platforms. Mol. Biol. Evol..

[23] Posada D. (2008). JModelTest: Phylogenetic model averaging. Mol. Biol. Evol..

[24] Ronquist F., Teslenko M., van der Mark P., Ayres D.L., Darling A., Höhna S., Larget B., Liu L., Suchard M.A., Huelsenbeck J.P. (2012). MrBayes 3.2: Efficient Bayesian phylogenetic inference and model choice across a large model space. Syst. Biol..

[25] Milutinović M., Petrović Z., Bobić B., Pavlović I. (1996). Ecological notes on ticks (Acari: Ixodidae) collected in west Serbia, Yugoslavia. Parasitol. Hung..

[26] Milutinović M., Masuzawa T., Tomanović S., Radulović Ž., Fukui T., Okamoto Y. (2008). *Borrelia burgdorferi sensu lato*, *Anaplasma phagocytophilum*, *Francisella tularensis* and their co-infections in host-seeking *Ixodes ricinus* ticks collected in Serbia. Exp. Appl. Acarol..

[27] Potkonjak A., Gutiérrez R., Savić S., Vračar V., Nachum-Biala Y., Jurišić A., Kleinerman G., Rojas A., Petrović A., Baneth G. (2016). Molecular detection of emerging tick-borne pathogens in Vojvodina, Serbia. Ticks Tick-Borne Dis..

[28] Tomanović S., Chochlakis D., Radulović Ž., Milutinović M., Ćakić S., Mihaljica D., Tselentis Y., Psaroulaki A. (2013). Analysis of pathogen co-occurrence in host seeking adult hard ticks from Serbia. Exp. Appl. Acarol..

[29] Tomanović S., Radulović Ž., Ćakić S., Mihaljica D., Sukara R., Penezić A., Burazerović J., Ćirović D. Tick species (acari: Ixodidae) of red foxes (*Vulpes vulpes*) in Serbia. Proceedings of the 2nd International Symposium on Hunting.

[30] Juwaid S., Sukara R., Penezić A., Mihaljica D., Veinović G., Kavallieratos N.G., Ćirović D., Tomanović S. (2019). First evidence of tick-borne protozoan pathogens, *Babesia* sp. and *Hepatozoon canis*, in red foxes (*Vulpes vulpes*) in Serbia. Acta Vet. Hung..

[31] Duscher G., Ćirović D., Heltai M., Szabó L., Lanszki J., Bošković I., Floriančić T., Knauer F., Suchentrunk F. (2014). Hepatozoonozis in golden jackal (*Canis aureus*) from southeastern and central Europe: Prevalence data from a first molecular screening. Book of Abstracts of First International Jackal Symposium.

[32] Banović P., Díaz-Sánchez A.A., Galon C., Foucault-Simonin A., Simin V., Mijatović D., Papić L., Wu-Chuang A., Obregón D., Moutailler S. (2021). A One Health approach to study the circulation of tick-borne pathogens: A preliminary study. One Health.

[33] Gabrielli S., Otašević S., Ignjatović A., Savić S., Fraulo M., Arsić-Arsenijević V., Momčilović S., Cancrini G. (2015). Canine babesioses in noninvestigated areas of Serbia. Vector-Borne Zoonotic Dis..

[34] Sukara R., Andrić N., Andrić J.F., Mihaljica D., Veinović G., Ranković V., Tomanović S. (2023). Autochthonous infection with *Ehrlichia canis* and *Hepatozoon canis* in dogs from Serbia. Vet. Med. Sci..

[35] Veinović G., Sukara R., Mihaljica D., Penezić A., Ćirović D., Tomanović S. (2024). The Occurrence and Diversity of Tick-Borne Pathogens in Small Mammals from Serbia. Vector-Borne Zoonotic Dis..

[36] Farkas R., Solymosi N., Takács N., Hornyák Á., Hornok S., Nachum-Biala Y., Baneth G. (2014). First molecular evidence of *Hepatozoon canis* infection in red foxes and golden jackals from Hungary. Parasites Vectors.

[37] Hodžić A., Georges I., Postl M., Duscher G.G., Jeschke D., Szentiks A.C., Ansorge H., Heddergott M. (2020). Molecular survey of tick-borne pathogens reveals a high prevalence and low genetic variability of *Hepatozoon canis* in free-ranging grey wolves (*Canis lupus*) in Germany. Ticks Tick Borne Dis..

[38] Battisti E., Zanet S., Khalili S., Trisciuoglio A., Hertel B., Ferroglio E. (2020). Molecular survey on vector-borne pathogens in alpine wild carnivorans. Front. Vet. Sci..

[39] Criado-Fornelio A., Martín-Pérez T., Verdú-Expósito C., Reinoso-Ortiz S.A., Pérez-Serrano J. (2018). Molecular epidemiology of parasitic protozoa and *Ehrlichia canis* in wildlife in Madrid (central Spain). Parasitol. Res..

[40] Ortuño M., Nachum-Biala Y., García-Bocanegra I., Resa M., Berriatua E., Baneth G. (2022). An epidemiological study in wild carnivores from Spanish Mediterranean ecosystems reveals association between *Leishmania infantum*, *Babesia* spp. and *Hepatozoon* spp. infection and new hosts for *Hepatozoon martis*, *Hepatozoon canis* and *Sarcocystis* spp. Transbound. Emerg. Dis..

[41] Helm S.C., Samson-Himmelstjerna V.G., Liesner M.J., Kohn B., Müller E., Schaper R., Pachnicke S., Schulze C., Krücken J. (2020). Identical 18S rRNA haplotypes of *Hepatozoon canis* in dogs and foxes in Brandenburg, Germany. Ticks Tick Borne Dis..

[42] Cardoso L., Cortes H.C., Eyal O., Reis A., Lopes A.P., Vila-Viçosa M.J., Rodrigues P.A., Baneth G. (2014). Molecular and histopathological detection of Hepatozoon canis in red foxes (*Vulpes vulpes*) from Portugal. Parasites Vectors.

[43] Dordio A.M., Beck R., Nunes T., Pereira da Fonseca I., Gomes J. (2021). Molecular survey of vector-borne diseases in two groups of domestic dogs from Lisbon, Portugal. Parasites Vectors.

[44] Criado-Fornelio A., Buling A., Pingret J.L., Etievant M., Boucraut-Baralon C., Alongi A., Agnone A., Torina A. (2009). Hemoprotozoa of domestic animals in France: Prevalence and molecular characterization. Vet. Parasitol..

[45] Giannelli A., Latrofa M.S., Nachum-Biala Y., Hodžić A., Greco G., Attanasi A., Annosica A., Otranto D., Baneth G. (2017). Three different Hepatozoon species in domestic cats from southern Italy. Ticks Tick Borne Dis..

[46] Baneth G., Mathew J.S., Shkap V., Macintire D.K., Barta J.R., Ewing S.A. (2003). Canine hepatozoonosis—Two disease syndromes caused by separate Hepatozoon species. Trends Parasitol..

[47] Ćirović D., Penezić A. (2019). Importance of slaughter waste in winter diet of wolves (*Canis lupus*) in Serbia. North-West. J. Zool..

[48] Kobryn H.T., Swinhoe E.J., Bateman P.W., Adams P.J., Shephard J.M., Fleming P.A. (2023). Foxes at your front door? Habitat selection and home range estimation of suburban red foxes (*Vulpes vulpes*). Urban Ecosyst..

[49] Lanszki J., Heltai M., Szabo L. (2006). Feeding habits and trophic niche overlap betw sympatric golden jackal (*Canis aureus*) and re (*Vulpes vulpes*) in the Pannonian ecoregion (Hungary). Can. J. Zool..

[50] Lainson R., Paperna I., Naiff R.D. (2003). Development of Hepatozoon caimani (Carini, 1909) Pess a, De Biasi & De Souza, 1972 in the Caiman *Caiman c. crocodilus*, the frog Rana catesbeiana and the mosquito *Culex fatigans*. Mem. Inst. Oswaldo Cruz..

[51] Viana L.A., Soares P., Silva J.E., Paiva F., Coutinho M.E. (2012). Anurans as paratenic hosts in the transmission of *Hepatozoon caimani* to caimans Caiman yacare and *Caiman latirostris*. Parasitol. Res..

[52] Sloboda M., Kamler M., Bulantová J., Votýpka J., Modrý D. (2008). Rodents as intermediate hosts of *Hepatozoon ayorgbor* (Apicomplexa: Adeleina: Hepatozoidae) from the African ball python, *Python regius*?. Folia Parasitol..

[53] Smith T.G., Desser S.S., Martin D.S. (1994). The development of *Hepatozoon sipedon* sp. nov. (Apicomplexa: Adeleina: Hepatozoidae) in its natural host, the Northern water snake (*Nerodia sipedon sipedon*), in the culicine vectors *Culex pipiens* and *C. territans*, and in an intermediate host, the Northern leopard frog (*Rana pipiens*). Parasitol. Res..

[54] Johnson E.M., Allen K.E., Panciera R.J., Ewing S.A., Little S.E. (2009). Experimental transmission of Hepatozoon americanum to New Zealand White rabbits (*Oryctolagus cuniculus*) and infectivity of cystozoites for a dog. Vet. Par..

[55] Johnson E.M., Panciera R.J., Allen K.E., Sheets M.E., Beal J.D., Ewing S.A., Little S.E. (2009). Alternate pathway of infection with Hepatozoon americanum and the epidemiologic importance of predation. J Vet. Intern. Med./ACVIM.

[56] Ćirović D., Penezić A., Milenković M., Paunović M., Hunting Association of Serbia (2008). Present distribution and factors of range spread of golden jackal (*Canis aureus* L. 1758) in Serbia. Proceedings of the International Conference on Large Carnivores, Žagubica, Serbia.

[57] Milenković M., Paunović M. (2003). Phenomenon of golden jackal (*Canis aureus* Linnaeus, 1758), expantion in Serbia. Carpathian Workshop on Large Carnivores Conservation. Convention on the Conservation of European Wildlife and Natural Habitats.

[58] Ćirović D., Penezić A., Plećaš M., Pokorny B., Kmetec U. (2018). Šakal (*Canis aureus*) u predelima sa dominantnim antropogenim uticajem. Slovenski lovski dan, Šakal v Sloveniji in Na Balkanu: Stanje in Upravljavski Izzivi.

[59] Hodžić A., Alić A., Prašović S., Otranto D., Baneth G., Duscher G.G. (2017). *Hepatozoon silvestris* sp. nov.: Morphological and molecular characterization of a new species of *Hepatozoon* (Adeleorina: Hepatozoidae) from the European wild cat (*Felis silvestris silvestris*). Parasitology.

