# Supplementary material for: Molecular Detection and Genetic Variability of Hepatozoon canis in Golden Jackals (Canis aureus L. 1758) in Serbia
Source: Biology (Basel). 2024 Jun 4;13(6):411. doi: 10.3390/biology13060411 (PMC11201132; doi:10.3390/biology13060411)
Supplement: Supplementary file 1 [file biology-13-00411-s001.zip › Table S1.pdf]

Table S1. List of sequences used in the present study

Acc number	Location	Year	Host/Vector
MH699890	Serbia	2013	*Vulpes vulpes*
PP711250	Serbia	2020	*Canis aureus*
MH699886	Serbia	2013	*Vulpes vulpes*
MH699888	Serbia	2013	*Vulpes vulpes*
MH699885	Serbia	2011	*Vulpes vulpes*
MH699892	Serbia	2011	*Vulpes vulpes*
MH699887	Serbia	2011	*Vulpes vulpes*
MH699891	Serbia	2013	*Vulpes vulpes*
MH699889	Serbia	2011	*Vulpes vulpes*
MH699884	Serbia	2010	*Vulpes vulpes*
OP012801	Serbia	2017	*Canis lupus*
OP012799	Serbia	2016	*Canis lupus*
PP711229	Serbia	2014	*Canis aureus*
PP711228	Serbia	2020	*Canis aureus*
PP711227	Serbia	2010	*Canis aureus*
PP711226	Serbia	2017	*Canis aureus*
PP711225	Serbia	2017	*Canis aureus*
PP711224	Serbia	2013	*Canis aureus*
PP711223	Serbia	2019	*Canis aureus*
PP711222	Serbia	2013	*Canis aureus*
PP711221	Serbia	2014	*Canis aureus*
PP711220	Serbia	2019	*Canis aureus*
PP711219	Serbia	2015	*Canis aureus*
PP711218	Serbia	2010	*Canis aureus*
PP711217	Serbia	2011	*Canis aureus*
PP711216	Serbia	2019	*Canis aureus*
OP012787	Serbia	2016	*Canis lupus*
OQ262961	Serbia	2011	*Apodemus flavicollis*
OP012778	Serbia	2012	*Canis lupus*
OP012782	Serbia	2011	*Canis lupus*
PP711230	Serbia	2014	*Canis aureus*
PP711231	Serbia	2020	*Canis aureus*
PP711232	Serbia	2013	*Canis aureus*
PP711233	Serbia	2014	*Canis aureus*
PP711234	Serbia	2020	*Canis aureus*
PP711236	Serbia	2017	*Canis aureus*
PP711245	Serbia	2013	*Canis aureus*
PP711246	Serbia	2013	*Canis aureus*
PP711244	Serbia	2020	*Canis aureus*
PP711237	Serbia	2013	*Canis aureus*
PP711238	Serbia	2013	*Canis aureus*
PP711239	Serbia	2019	*Canis aureus*
PP711240	Serbia	2013	*Canis aureus*
PP711241	Serbia	2017	*Canis aureus*

PP711242	Serbia	2020	*Canis aureus*
MZ146329	Serbia	2019	*Rhipicephalus sanguineus*
OP012788	Serbia	2016	*Canis lupus*
OP012794	Serbia	2012	*Canis lupus*
PP711247	Serbia	2017	*Canis aureus*
PP711249	Serbia	2020	*Canis aureus*
PP711243	Serbia	2010	*Canis aureus*
PP711235	Serbia	2020	*Canis aureus*
OP012777	Serbia	2012	*Canis lupus*
OP012798	Serbia	2017	*Canis lupus*
PP711248	Serbia	2010	*Canis lupus*
OP012790	Serbia	2014	*Canis lupus*
OP012800	Serbia	2017	*Canis lupus*
OP012779	Serbia	2017	*Canis lupus*
OP012792	Serbia	2016	*Canis lupus*
OP012775	Serbia	2014	*Canis lupus*
OP012773	Serbia	2014	*Canis lupus*
OP012783	Serbia	2019	*Canis lupus*
OP012793	Serbia	2012	*Canis lupus*
OP012781	Serbia	2014	*Canis lupus*
OP012780	Serbia	2014	*Canis lupus*
OP012791	Serbia	2017	*Canis lupus*
OP012797	Serbia	2012	*Canis lupus*
OP012802	Serbia	2010	*Canis lupus*
OP012774	Serbia	2011	*Canis lupus*
OP012776	Serbia	2018	*Canis lupus*
OP012795	Serbia	2016	*Canis lupus*
OP012786	Serbia	2014	*Canis lupus*
OP012789	Serbia	2015	*Canis lupus*
OP012785	Serbia	2014	*Canis lupus*
OP012784	Serbia	2015	*Canis lupus*
OP012796	Serbia	2017	*Canis lupus*
MZ930460	Serbia	2018	*Canis lupus familiaris*
