# Supplementary figures and images for: Molecular Detection and Genetic Variability of Hepatozoon canis in Golden Jackals (Canis aureus L. 1758) in Serbia
Source: Biology (Basel). 2024 Jun 4;13(6):411. doi: 10.3390/biology13060411 (PMC11201132; doi:10.3390/biology13060411)

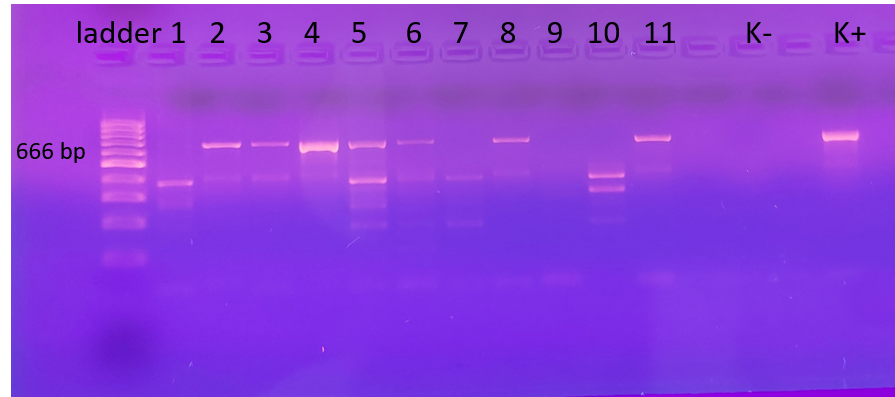

Supplement: Supplementary file 1 [file biology-13-00411-s001.zip › Figure S1.tif]
